# The fat-mass and obesity-associated gene rs9939609 T allele is prominent among the native Mexican population and is associated with risk for Type 2 diabetes and metabolic dysfunction-associated steatotic liver disease

**DOI:** 10.3389/fnut.2025.1569342

**Published:** 2025-10-10

**Authors:** Maricruz Sepulveda-Villegas, Arturo Panduro, Leonardo Leal-Mercado, Juan Pablo Cardenas-Benitez, Claudia Ojeda-Granados, Sonia Roman

**Affiliations:** ^1^Department of Genomic Medicine in Hepatology, Civil Hospital of Guadalajara, “Fray Antonio Alcalde”, Guadalajara, Jalisco, Mexico; ^2^Health Sciences Center, University of Guadalajara, Guadalajara, Jalisco, Mexico; ^3^Programa Doctoral en Biología Molecular en Medicina, Health Sciences Center, University of Guadalajara, Guadalajara, Jalisco, Mexico

**Keywords:** *FTO*, Amerindian, type 2 diabetes, MASLD, metabolic syndrome, dyslipidemia, obesity, ancestry

## Abstract

**Background:**

The fat mass and obesity-associated (*FTO*) rs9939609 T>A polymorphism is associated with excess body fat and metabolic disturbances, including Type 2 diabetes (T2D) and metabolic dysfunction-associated steatotic liver disease (MASLD). However, the genetic effect of the T and the A alleles on the development of these diseases may vary among populations.

**Objective:**

This study aimed to determine the distribution of the *FTO* rs9939609 T>A polymorphism in West Mexican populations with variable genetic ancestry and analyze its effect on an admixed cohort’s anthropometric and metabolic profile.

**Methods:**

In a cross-sectional study, 684 unrelated adults from West Mexico were included. Subjects were grouped as Amerindian (Wixárika and Nahuas) or admixed: Mestizo-Caucasians (Cuquío, San Miguel-Los Altos, and Villa Purificación) and Mestizo-Guadalajara (Mestizo-GDL). *FTO* genotyping was determined by an allelic discrimination assay. Assessment of anthropometrics, diet composition, and metabolic profile among 333 Mestizo-GDL subjects and their association with metabolic risk factors was conducted considering the dominant model (AA + AT vs. TT).

**Results:**

The Wixárika group had the highest frequencies of the T allele (94%) and TT genotype (89%) among Amerindians, followed by mestizos from GDL (74% and 56%, respectively). In contrast, Mestizo-Caucasians from Cuquío had the highest A allele frequency (32.4%). No significant effect of the FTO genotype on body mass index (BMI)/body fat was observed in the Mestizo-GDL population. However, the TT carriers exhibited higher waist-to-height ratios (0.52 ± 0.07 vs. 0.49 ± 0.08), insulin levels (10.8 *±* 7.3 vs. 8.8 ± 5.2 μU/mL), triglycerides (141.8 ± 66.5 vs. 125.8 ± 65.3 mg/dL), and VLDL-c (29.1 ± 14.8 vs. 25.6 ± 14.2 mg/dL) than AA + AT carriers. The TT genotype was associated with higher odds of hypertriglyceridemia (HTG) (OR = 1.7, 95% CI = 1.07–2.73, *p* = 0.027), insulin resistance (IR) (OR = 1.79, 95% CI = 1.06–3.07, *p* = 0.031), and hyperglycemia (HGL) (OR = 2.77, 95% CI = 1.5–5.36, *p* = 0.002). Multivariable logistic regression confirmed that TT genotype carriers had greater odds of HGL (OR = 2.50, 95% CI = 1.2–5.15, *p* = 0.013).

**Conclusion:**

The *FTO* T allele was prominent among native Mexicans. In contrast, the A allele prevailed among the Mestizo with higher European ancestry. The TT genotype carriers had higher odds of IR, HTG, and HGL, highlighting the genetic predisposition to T2D and MASLD in populations exposed to obesogenic and hepatopathogenic environments.

## 1 Introduction

Excess visceral adipose tissue, or abdominal obesity, is a significant risk factor for metabolic abnormalities such as hypoalphalipoproteinemia (HALP), hypertriglyceridemia (HTG), hypercholesterolemia (HCL), insulin resistance (IR), hyperglycemia (HGL), and elevated levels of liver enzymes, alanine aminotransferase (ALT), and aspartate aminotransferase (AST). Most of these obesity-related conditions are key triggers of Type 2 diabetes mellitus (T2D), cardiovascular disease, and non-alcoholic fatty liver disease (1–3), now referred to as metabolic dysfunction-associated steatotic liver disease (MASLD) (4).

Globally, the number of women and men with obesity in 2022 was 504 million and 374 million, respectively, which represents an increase of 684 million people since 1990 (5). Countries experiencing a nutrition transition tend to have the highest rates of obesity, T2D, and MASLD (6–9). Currently, in Mexico, 74% of the population has excess body weight, of which 4% presents class III obesity (10). Consequently, a significant portion of Mexicans are at risk for various health conditions. Estimates from the 2022 National Survey of Health and Nutrition indicate a prevalence of 22.1% of prediabetes and 18.3% of T2D (11). In a recent study carried out in West Mexico, 57% of 505 patients were at risk for MASLD (12). These chronic conditions rank within the top five causes of morbidity in Mexico (13) and are caused by intricate interactions between obesogenic environments (including hepatopathogenic foods, sedentary lifestyles, and stress) and genetic risk factors, which may vary by region.

The fat mass and obesity-associated (*FTO*) gene, originally named “Fatso” due to its abbreviation from “FT” (short for “Fused Toes”), referring to limb deformities observed in rats lacking this gene (14), has attracted renewed attention in recent years due to its association with weight gain. Genome-wide Association Studies have identified *FTO* as the first obesity-susceptibility gene in populations of European ancestry, resulting in its renaming as the “fat mass and obesity-associated” gene (15, 16). The *FTO* gene (16q12.2) encodes an alpha-ketoglutarate-dependent dioxygenase that performs oxidative demethylation, modifying nucleic acids (DNA and RNA). This enzyme has biological activity in a variety of tissues, predominantly in the brain, regulating the hunger/satiety axis and reward system; in the liver, controlling the expression of lipogenic genes and cellular proliferation; and in adipocytes, influencing the size of white adipose tissue, cell differentiation, browning, and thermogenesis (17, 18).

The *FTO* gene has been extensively studied for its implications in energy balance (19–21), stimulating adipogenesis, and promoting lipid storage in mature adipocytes (22–24). This gene is highly polymorphic, and among the most studied single-nucleotide polymorphisms (SNP) is the *FTO* rs9939609 T>A variant in which the highest frequency of the risk A allele has been found in African (49%) and European populations (45%) (25). The presence of the A allele increases *FTO* gene expression and adipocyte size, which results in a lower quantity of mitochondria (26). Each copy of the A allele is associated with increased BMI by approximately 0.4 kg/m^2^ and body weight by 1.2 kg in adults (15). Clinical research indicates that the A allele is linked to lower satiety, overeating, a preference for energy-dense foods, lower resting energy expenditure, and a higher prevalence of obesity and severe obesity in Caucasian populations (26–28).

Several studies have reported the association between the A allele and elevated BMI in Mexican subpopulations differing by geographic locations and genetic ancestries (29–32). This ancestral genetic diversity is distinguished by variable degrees of Amerindian, European, and African lineages, considerably influencing several biological characteristics, including anthropometric and metabolic phenotypes (33–35). Furthermore, in prior research, we reported a differential distribution of the genetic and phenotypic traits associated with dyslipidemias across Mexican subpopulations of West Mexico (36, 37) that have been linked to the increased prevalence of IR, HTG, HALP, HCL, and HGL in at-risk individuals for MASLD (12).

Considering the role of *FTO* in weight gain and the genetic heterogeneity of the Mexican population, it is plausible that the respective T and the A alleles could differentially influence the anthropometric and metabolic profile linked to the risk for T2D and MASLD. Therefore, this study aimed to determine the distribution of the *FTO* rs9939609 T>A polymorphism in West Mexican populations with variable genetic ancestry and analyze its effect on an admixed cohort’s anthropometric and metabolic profile.

## 2 Materials and methods

### 2.1 Participants and study design

This cross-sectional study involved 684 adult participants of both genders from different regions of the State of Jalisco and Nayarit in Western Mexico. The distribution of the *FTO* (rs9939609) polymorphism among participants was determined using a convenience sampling method. The study population was stratified into three groups according to their prominent genetic ancestry, as described previously (38, 39). Briefly, Wixárika (also known as Huichol) (*n* = 100) and Nahuas (*n* = 84) with a background of Amerindian history; Mestizo-Guadalajara (Mestizo-GDL) (*n* = 333) are admixed populations with an intermediate Amerindian-European ancestry, and Mestizo with major European ancestry were denoted as Mestizo-Caucasians (towns of Cuquío, *n* = 102; San Miguel-Los Altos, *n* = 33; Villa Purificación, *n* = 32).

The recruitment process for the Amerindian groups took place at their rural medical service following an invitation placed by their tribal chief. Mestizo-Caucasian participants were recruited at their community health centers by a public invitation.

The sample size for association analyses in the Mestizo-GDL population was calculated using the formula for quantitative variables in comparative studies, and was based on the allele frequency of the *FTO* (rs9939609) polymorphism in a case (obese)-control (normal weight) study in Mexicans reported by Villalobos-Comparán et al. (29). The Mestizo-GDL group was outpatients attending the Nutrigenetic Clinic of the Department of Genomic Medicine in Hepatology, Civil Hospital of Guadalajara “Fray Antonio Alcalde.” Participants eligible for inclusion were adults with a BMI ≥18.5 kg/m^2^ who self-reported as healthy and had no prior medical diagnosis of metabolic dysfunction-associated steatotic liver disease, T2DM, or other chronic conditions, and were not currently receiving treatment for any metabolic disorder. Exclusion criteria comprised pregnancy or lactation, a history of excessive alcohol consumption (>20 g/day for females and >40 g/day for males), confirmed hepatitis B or C virus infection, or evidence of drug-induced hepatotoxicity. The study was conducted from February 2018 to December 2019 and was approved by the Institutional Board Committee (#CI-07218). All participants signed an informed consent before the study.

### 2.2 Genotyping analysis

Genomic DNA was extracted from leucocytes by a modified salting-out method (40). *FTO* rs9939609 T>A polymorphism genotyping was performed using a predesigned TaqMan allelic discrimination assay (C_30090620_10, Applied Biosystems, Foster, CA, United States) by a Real-Time PCR technique running on Step One Plus thermocycler (Applied Biosystems, Foster, CA, United States). DNA concentration per sample and assay was 20 ng/μL. PCR conditions were enzyme activation at 95 °C for 10 min, followed by 40 cycles of denaturation at 95 °C for 15 s and annealing/extension at 60 °C for 1 min. Genotyping concordance was verified by including a positive control corresponding to the three possible genotypes in the run. Genotyping was repeated on 10 random samples per plate as a quality control check. The allelic and genotypic distribution of the *FTO* rs9939609 T>A polymorphism was determined among the population groups by the simple count method.

### 2.3 Anthropometric and dietary intake assessment

Height was measured without shoes to the nearest 0.1 cm using a stadiometer (Rochester Clinical Research, NY, United States). Body circumferences were measured using a steel flexible tape (Ross Craft Anthrotape, Rosscraft Innovations Inc., Toronto, Canada). The waist circumference measurement was taken at the midpoint between the iliac crest and the edge of the last rib on a horizontal plane, with an accuracy of 0.1 cm. Hip circumference was measured at the maximum protuberance of the gluteus on a horizontal plane, with an accuracy of 0.1 cm. Body composition was determined by electrical bioimpedance (InBody 3.0 Analyzer Composition, and Bio Space, Gangnam-gu, Seoul, Korea) and classified by BMI using the WHO criteria (41). Waist-to-height ratio (WHtR) was determined by the waist circumference divided by the individual’s height, measured in centimeters.

Diet was assessed using a three-day dietary record (two weekdays and one weekend). Average energy and nutrient intake data were processed using Nutrikcal software (Nutrikcal VO^®^, Mexico City, Mexico), which considers the nutritional composition database of Mexican foods (42).

### 2.4 Biochemical profile

A 10-mL blood sample was obtained via venipuncture after a 12-h overnight fast to assess the participants’ biochemical profile, which included glucose, insulin, triglycerides (TG), total cholesterol (TC), high-density lipoprotein cholesterol (HDL-c), AST, and ALT. All biochemical tests were conducted using the Clinical Chemistry System (Beckman Coulter’s Inc., California, United States). Low-density lipoprotein cholesterol (LDL-c) concentration was determined using the Friedewald formula (43). Very low-density lipoprotein cholesterol (VLDL-c) concentration was calculated by subtracting the sum of LDL-c and HDL-c from total cholesterol.

IR was evaluated through the homeostatic model assessment (HOMA-IR) using the equation (fasting glucose mg/dL × fasting serum insulin μU/mL)/405 (44). Dyslipidemias were defined according to the NCEP-ATP III (National Cholesterol Education Program Adult Treatment Panel III) as follows: HCL as TC ≥200 mg/dL, high LDL-c (H-LDL) as LDL-c ≥130 mg/dL, HALP as HDL-c <40 mg/dL, and HTG as TG ≥150 mg/dL (45). In addition, HGL was defined as fasting glucose ≥100 mg/dL (45), hyperinsulinemia (HINS) as fasting insulin >9 μU/mL (46), and IR as HOMA-IR ≥2.5 (44).

### 2.5 Statistical analysis

Qualitative traits were expressed as numbers and percentages, and quantitative traits as mean ± standard deviation (SD). Chi-square test was used to assess the differences between qualitative variables, and the allelic and genotypic frequencies between the study groups. The normal distribution of quantitative variables was evaluated using the Kolmogorov–Smirnov test. Accordingly, the Student’s *t*-test or Mann–Whitney *U* test evaluated significant differences between variables. The difference in dietary intake between the *FTO* rs9939609 genotypes was analyzed with the ANCOVA test adjusted for energy intake. It has been reported that the genetic effect of the *FTO* rs9939609 T>A polymorphism is additive (46); however, given the low prevalence of the AA genotype in the study population, the analyses based on the genotype were performed using the dominant genetic model (i.e., AA + AT vs. TT). Bivariate and BMI-adjusted multivariable logistic regression analyses were used to evaluate the association between *FTO* rs9939609 genotypes (AA + AT vs. TT) and metabolic abnormalities. Results were expressed as odds ratio (OR) with a 95% confidence interval (CI). A *p*-value <0.05 was considered significant. The statistical analysis was performed with SPSS software (version 20.0; SPSS Inc., Chicago, IL, United States). Hardy–Weinberg equilibrium (HWE) was analyzed with Arlequin software for Windows (version 3.1; Berne, Switzerland).

The principal component analysis (PCA) was implemented to plot the genetic divergence between the study populations. Reference populations EUR_TSI (Tuscans, Italy); EUR_CEU (Central Europe); EAS_JPT (East Japan, Tokyo) were included to analyze genetic differentiation and ancestral components based on the genotypic frequencies for the *FTO* rs9939609 T>A polymorphism (1,000 Genomes Project, http://www.1000genomes.org/). Other Mexican study groups’ *FTO* rs9939609 allele frequency data were also included (Supplementary Table S1). The R programming language within the R Studio environment was used for the PCA (ver 4.3.3.).

## 3 Results

### 3.1 Distribution of the *FTO* rs9939609 polymorphism and PCA analysis

Table 1 depicts the distribution of the *FTO* rs9939609 T>A polymorphism among the West Mexico subpopulations included in this study. The Wixárika population had the highest T allele and TT genotype frequencies (94% and 89%), followed by the Mestizo-GDL (74% and 56%). In contrast, Mestizo-Caucasians from the town of Cuquío had the highest A allele frequency (32.4%).

**Table 1:** Allelic and genotypic distribution among the study population of West Mexico.

		Ancestry					
	Total	Amerindian		Mestizo-GDL	Mestizo-Caucasians		
	*n* = 684	Wixárika *n* = 100	Nahuas *n* = 84	Guadalajara *n* = 333	Cuquío *n* = 102	Los Altos *n* = 33	Villa P *n* = 32
Alleles (*n*, %)							
A	160 (23)	6 (6)	14 (16.7)	88 (26)	33 (32.4)	9 (27.2)	9 (28.1)
T	524 (77)	94 (94)^*^	70 (83.3)	245 (74)	69 (67.6)	24 (71.8)	23 (71.9)
Genotypes (*n*, %)							
AA	54 (8)	1 (1)	4 (4.8)	28 (8)	13 (12.7)	3 (9.1)	5 (15.6)
AT	211 (31)	10 (10)	20 (23.8)	120 (36)	40 (39.2)	13 (39.4)	8 (25)
TT	419 (61)	89 (89)^*^	60 (71.4)	185 (56)	49 (48)	17 (51.5)	19 (59.4)
HWE	—	0.296	0.228	0.159	0.364	1.000	0.156

Data is presented as number (*n*) and percentage (%). Differences in allelic and genotypic frequencies between populations were analyzed using the chi-square test. ^*^Wixárika vs. all groups *p* = 0001. HWE, Hardy–Weinberg equilibrium; Villa P, Villa Purificación; A, adenine; T, thymine.

**Figure 1:**
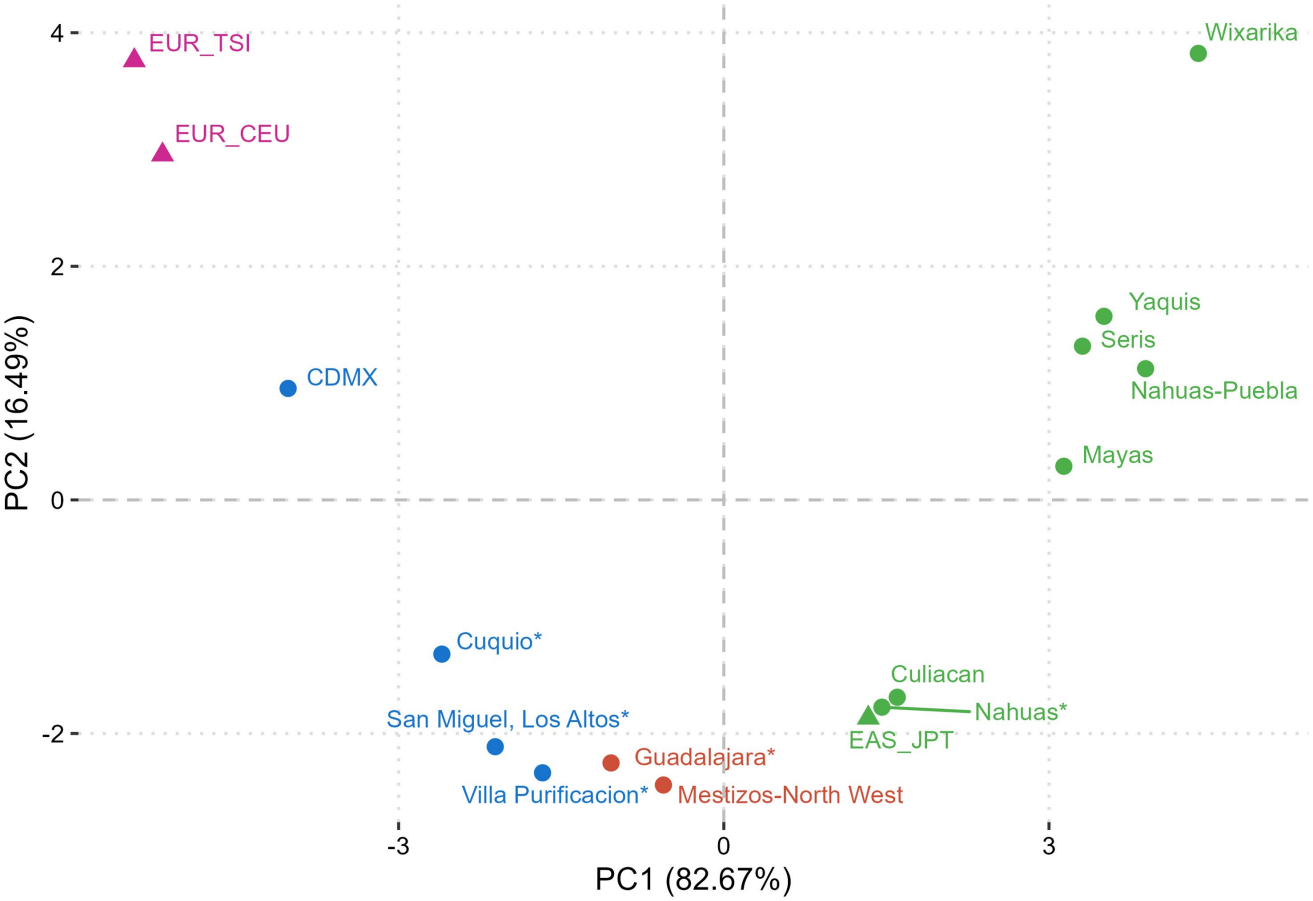
Principal component analysis (PCA) of ancestrality based on the *FTO* rs9939609 polymorphism. Populations with similar genetic components clustered based on their genetic distances. Mexican Natives (Nahuas* and Wixárika*) grouped with Amerindian references, Mestizo-Caucasians (Cuquío*, San Miguel-Los Altos*, Villa Purificación*) clustered with higher European ancestry study groups, and Mestizo-GDL (Guadalajara*) with intermediate ancestry clustered with Mestizo-Northwest. *This study. Non-Mexican population references: EUR_TSI (Tuscans, Italy); EUR_CEU (Central Europe); EAS_JPT (East Japan, Tokyo) from 1,000 Genomes Project. Mexican population references: CDMX (29), Mestizo-Northwest (48), Culiacan (66), Mayas (49), Nahuas-Puebla (48), Seris (48), and Yaquis (48). Purple dots: European ancestry; Blue dots: Admixed ancestry. Green dots: Amerindian ancestry.

Figure 1 illustrates the genetic differentiation between the study and reference populations in agreement with this *FTO* allelic/genotypic distribution. The Wixárika (Huichol) were the most differentiated group among the Amerindian clusters, which contained data from the north (Yaquis, Seris), central (Nahuas-Puebla), and south (Mayas) of Mexico, and the Nahuas study group. The Cuquío, San Miguel-Los Altos, and Villa Purificación populations clustered towards the EUR_TSI, EUR_CEU, and CDMX reference groups. In contrast, Mestizo-GDL formed an intermediate cluster with Mestizo-Northwest in agreement with the reported ancestry of these populations.

### 3.2 Association of the *FTO* rs9939609 T>A polymorphism with demographic, anthropometric, and biochemical characteristics of Mestizo-GDL

In the Mestizo-GDL group, anthropometric, dietary intake, and biochemical profile assessments were performed, and their association with the *FTO* rs9939609 polymorphism was analyzed. The clinical data of the Mestizo-GDL were analyzed by genotypes (AA + AT vs. TT) (Table 2). The mean age of the subjects was 36.4 ± 12.9 years, and the frequency of genotypes was 46% for AA + AT and 56% for TT. No significant differences were observed in age, gender, BMI, body fat percentage, HOMA-IR, TC, HDL-c, LDL-c, AST, and ALT between AA + AT and TT genotype carriers. Nevertheless, TT genotype carriers presented significantly higher mean values of WHtR (0.52 ± 0.070 vs. 0.49 ± 0.08), insulin (10.8 ± 7.3 μU/mL vs. 8.8 ± 5.2 μU/mL), TG (141.8 ± 66.5 mg/dL vs. 125.8 ± 65.3 mg/dL), and VLDL-c (29.1 ± 14.8 mg/dL vs. 25.6 ± 14.2 mg/dL) compared to AA + AT genotype carriers (*p* < 0.05).

**Table 2:** Demographic, anthropometric, and biochemical characteristics of the Mestizo-GDL population.

Variables	Total	AA + AT	TT	*p*-value
Subjects, *n* (%)	333 (100)	148 (44)	185 (56)	—
Age (years)	36.4 ± 12.9	35 ± 12.8	37.6 ± 12.9	0.061
Gender (F/M)	248/85	117/31	131/54	0.085
BMI (kg/m^2^)	28.3 ± 7.9	28.1 ± 8.1	28.4 ± 7.8	0.322
Body fat (%)	30.9 ± 9.2	30.3 ± 9	31.4 ± 9.4	0.327
Waist-to-height ratio	0.51 ± 0.08	0.49 ± 0.08	0.52 ± 0.07	<0.001
Glucose (mg/dL)	91.3 ± 18.6	88.7 ± 11.3	93.5 ± 22.6	0.065
Insulin (μU/mL)	9.9 ± 6.5	8.8 ± 5.2	10.8 ± 7.3	0.041
HOMA-IR	2.2 ± 1.8	1.9 ± 1.3	2.4 ± 2.1	0.178
TG (mg/dL)	134.6 ± 66.3	125.8 ± 65.3	141.8 ± 66.5	0.017
TC (mg/dL)	186.3 ± 38.6	182.4 ± 32.1	189.4 ± 42.8	0.092
HDL-c (mg/dL)	44.1 ± 12.6	45.8 ± 12.9	42.9 ± 12.2	0.085
LDL-c (mg/dL)	113.8 ± 31.6	111.2 ± 26	115.9 ± 35.4	0.221
VLDL-c (mg/dL)	27.5 ± 14.6	25.6 ± 14.2	29.1 ± 14.8	0.015
AST (IU/L)	27.4 ± 11.5	26 ± 10.7	28.4 ± 12	0.075
ALT (IU/L)	27.7 ± 15.6	26 ± 12.8	29.1 ± 17.5	0.201

Average values are expressed as mean ± SD. Data comparison between genotypes was performed using the Student’s *t* test or Mann–Whitney *U* test as appropriate. *n*, number; F, female; M, male; BMI, body mass index; HOMA-IR, homeostasis model assessment of insulin resistance; TG, triglycerides; TC, total cholesterol; HDL-c, high density lipoprotein cholesterol; LDL-c, low density lipoprotein cholesterol; VLDL-c, very low-density lipoprotein cholesterol; AST, aspartate aminotransferase; ALT, alanine aminotransferase.

### 3.3 Association of *FTO* rs9939609 T>A polymorphism genotypes with metabolic abnormalities in Mestizo-GDL

**Figure 2:**
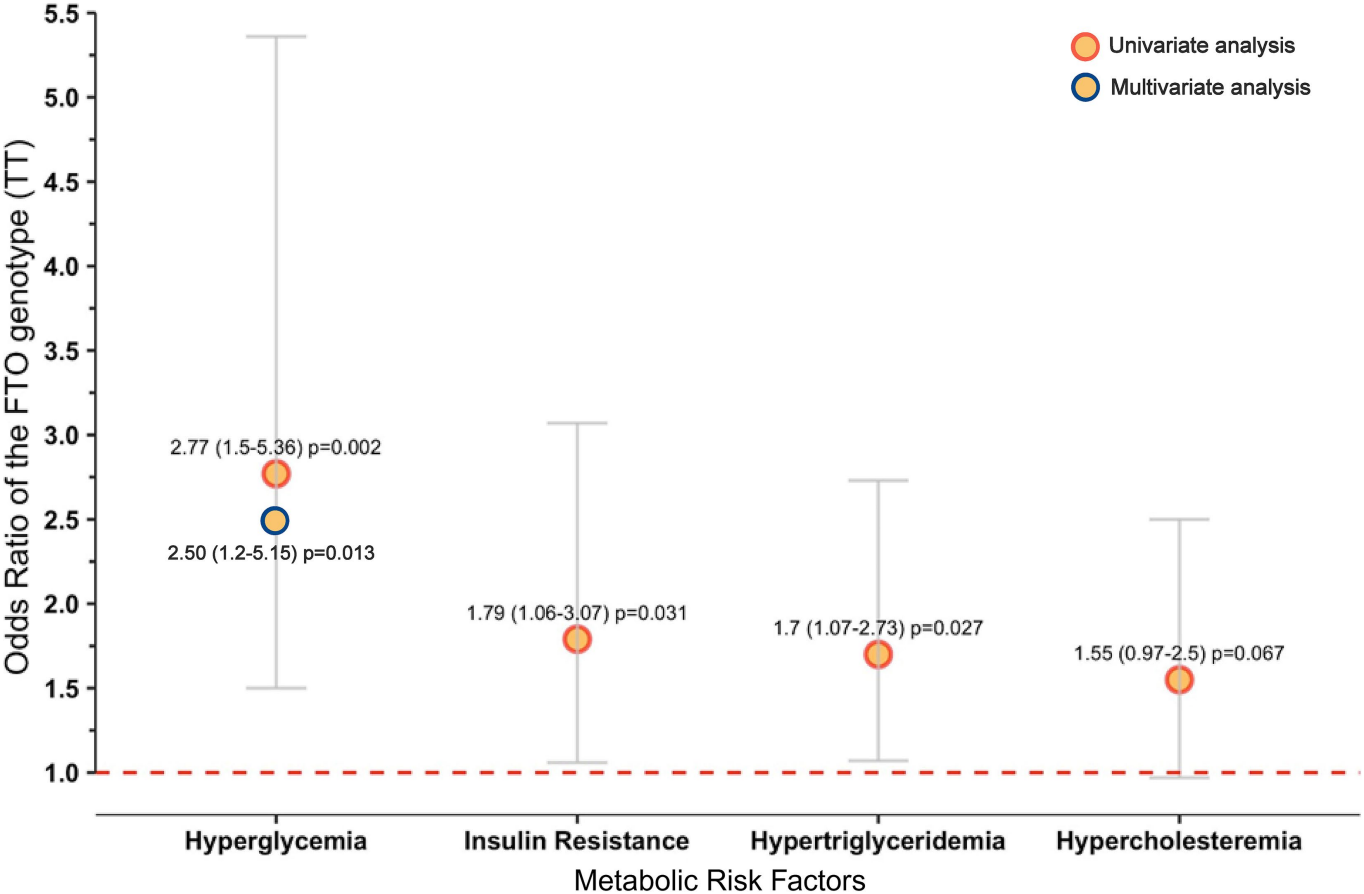
Metabolic abnormalities associated with the TT genotype of the *FTO* rs9939609 polymorphism in the Mestizo-GDL population. Hosmer and Lemeshow test: chi-square = 1.497, *p* = 0.473. Only variables with *p* < 0.05 from the univariate analysis were introduced in the multivariable analysis. Definitions: OR (95% CI), odds ratio (95% confidence interval). Hyperglycemia was defined as fasting glucose levels ≥100 mg/dL, insulin resistance as HOMA-IR ≥ 2.5, hypertriglyceridemia as triglyceride levels ≥150 mg/dL, and hypercholesterolemia as total cholesterol levels ≥200 mg/dL.

Bivariate and BMI-adjusted multivariable logistic regression analysis was performed to assess the probability of metabolic abnormalities associated with the genotypes of the *FTO* rs9939609 T>A polymorphism among the Mestizo-GDL. In the bivariate analysis, HGL, HOMA-IR, and HTG were related to the TT genotype (*p* < 0.05) (Figure 2). According to multivariable analysis, individuals with the TT genotype had a 2.5-fold higher OR of having HGL (95% CI 1.213–5.152, *p* = 0.013) than carriers of the AA + AT genotypes (Figure 2).

### 3.4 Dietary composition analysis of the study population adjusted by genotypes of the *FTO* rs9939609 polymorphism

Considering that dietary patterns can influence biochemical profiles, the nutritional characteristics of Mestizo-GDL subjects were analyzed according to *FTO* rs9939609 genotypes (AA + AT vs. TT) (Table 3). No significant differences in energy and nutrient intake by genotype were observed. Similarly, no significant differences were found when analyzing whether being a carrier of the T allele affected total kilocalories or impacted nutrient intake.

**Table 3:** Dietary intake adjusted by *FTO* rs9939609 genotypes among the Mestizo-GDL group.

Variables	Total	AA + AT	TT	*p*-value
Subjects, *n* (%)	333 (100)	148 (44)	185 (56)	—
Energy intake, kcal	2072.4 ± 799.4	2111.8 ± 923.1	2040.9 ± 685.6	0.578
Proteins, %	16.8 ± 4	16.9 ± 4.1	16.7 ± 3.9	0.573
Lipids, %	33.7 ± 9.1	33.3 ± 9.1	34 ± 9.2	0.441
Saturated fatty acids, g	22 ± 13.4	22.8 ± 14.7	21.3 ± 12.3	0.514
Monounsaturated fatty acids, g	25.7 ± 16.2	26.2 ± 19.2	25.2 ± 13.3	0.971
Polyunsaturated fatty acids, g	11.7 ± 8.3	11.9 ± 9.8	11.5 ± 7	0.894
Dietary cholesterol, mg (Median, Q1, Q3)	244.0 (165.0, 358.0)	229.5 (150.7, 318.5)	263.0 (170.0, 391.0)	0.064
Carbohydrates, %	50.8 ± 9.6	51.6 ± 10	50.2 ± 9.3	0.188

Values are expressed as mean ± SD unless expressed otherwise. Differences between energy and nutrient intake were tested by chi-square, Student’s *t*, or Mann–Whitney tests according to the variable type (qualitative or quantitative). ANCOVA tests were adjusted by energy intake. *n*, number; %, percentage; kcal, kilocalories.

## 4 Discussion

This study reports the distribution of the *FTO* rs9939609 T>A polymorphism in West Mexican populations with different proportions of genetic ancestral components. A higher frequency of the wild-type or major T allele versus the A risk allele was found in Amerindian populations, particularly in the Wixárika (T = 0.94/A = 0.06), the highest frequency reported to date (47). These findings are consistent with earlier studies revealing a higher T vs. A allele frequency among Amerindians throughout Mexico. For example, the T/A allele proportion among the Yaquis and Seris of North Mexico ranges from 0.86/0.14 to 0.91/0.09, respectively (29, 48). Other Amerindian groups follow the same pattern, such as the Nahuas from Central Mexico (0.94/0.06) (31), Zapotecas in southwest Oaxaca (0.99/0.01) (29), and Mayas in southeast Mexico (0.88/0.12) (49). In this study, the Mestizo-GDL characterized by an intermediate proportion of Amerindian and European ancestries as shown by the PCA, revealed a T/A proportion of 0.74/0.26, similar to other studies in Mexican-Mestizo (48), followed by those with a higher European ancestry (Mestizo-Caucasians) with a T/A average ratio of 0.70/0.30. Furthermore, this latter subgroup formed a cluster diverging from the Mestizo-GDL and Amerindians, consistent with these groups’ genetic and demographic history as previously reported (50). These results reflect the overall gradient of the European–Amerindian ancestral components encountered among admixed Mexicans across the country (33, 35, 51).

Earlier studies in the Mexican population have reported an association of the A allele with the risk of class III obesity (29–32) and emotional undereating or food preferences (49, 52, 53). This study found no significant differences in BMI or body fat percentage among the genotype (AA + AT vs. TT) categories. Nonetheless, carriers of the TT genotype exhibited significantly higher WHtR values, a reliable indicator of central obesity and cardiometabolic risk (54). In addition, these subjects had higher insulin, TG, and VLDL-c levels compared to AA + AT carriers. Furthermore, the bivariate analysis revealed a significant association between the TT genotype and HGL, IR, and HTG.

On the other hand, the multivariable analysis revealed that TT genotype carriers were 2.5-fold more likely to have HGL than AA + AT carriers, irrespective of their BMI. This finding may be unexpected since the A allele has been associated with increased risk, particularly of obesity, in various populations worldwide (55). Notably, the T allele or TT genotype has been associated with a leaner phenotype and better performance in energy sports among elite athletes (56).

As reported previously, the pre-Hispanic, traditional Mexican diets were historically rich in fiber, including soluble and insoluble fiber. These diets primarily consisted of endemic fruits and vegetables, particularly those found in the “Milpa” system (beans, maize, chili, zucchini, tomato). They were also abundant in minerals, vitamins with antioxidant properties, and bioactive compounds while being high in monounsaturated and polyunsaturated fats and low in saturated fats (36). However, the recent shift towards a diet rich in saturated fats and sugars, as shown in the study group, has been related to an evolutionary mismatch (57, 58). Although in this study, no significant differences in diet composition between the AA + AT and TT genotypes were observed, their dietary profiles were unhealthy, with high intakes of total fat, saturated fat, and simple carbohydrates. This dietary pattern is consistent with the “hepatopathogenic and obesogenic” features previously described in the West Mexican population, regardless of BMI (12). Furthermore, the metabolic abnormalities found in Mestizo-GDL subjects, particularly in carriers of the TT genotype, are components of the metabolic syndrome, a significant risk factor for developing cardiovascular disease, T2D, and MASLD (59). Therefore, replacing the traditional diet with contemporary hepatopathogenic foods, coupled with the presence of risk alleles, including the *FTO* rs9939609 polymorphism, could contribute to the higher susceptibility for dyslipidemias, IR, and HGL among the Mexican population (58, 60, 61).

Conventionally, the minor A allele (rs99396009) is the most widely studied in Mexico and globally associated with the risk for excess weight, body fat, and extreme obesity, as mentioned before. However, less attention has been given to the major T allele and its impact on metabolic health in at-risk populations, which were highly prevalent in this study. The findings in this study could be explained by considering the molecular role of the *FTO* gene, particularly in lipid metabolism, adipocyte maturation (20–22), and the adipose tissue expandability hypothesis (62). When the adipose capacity is exceeded due to excess caloric intake, the excess energy is stored in non-adipose tissues, favoring lipotoxicity and metabolic disturbances such as dyslipidemia and IR (62–64). A lower expression of *FTO* in TT genotype carriers has been reported (29, 30), which could contribute to decreased adipogenesis and limited energy storage by adipocytes (65), compared to AA genotype carriers, in which elevated adipose storage or obesity is observed (15, 55). Subjects carrying the AA + AT compared to TT genotypes might have different adipose cell storage capacities in response to excess energy intake.

**Figure 3:**
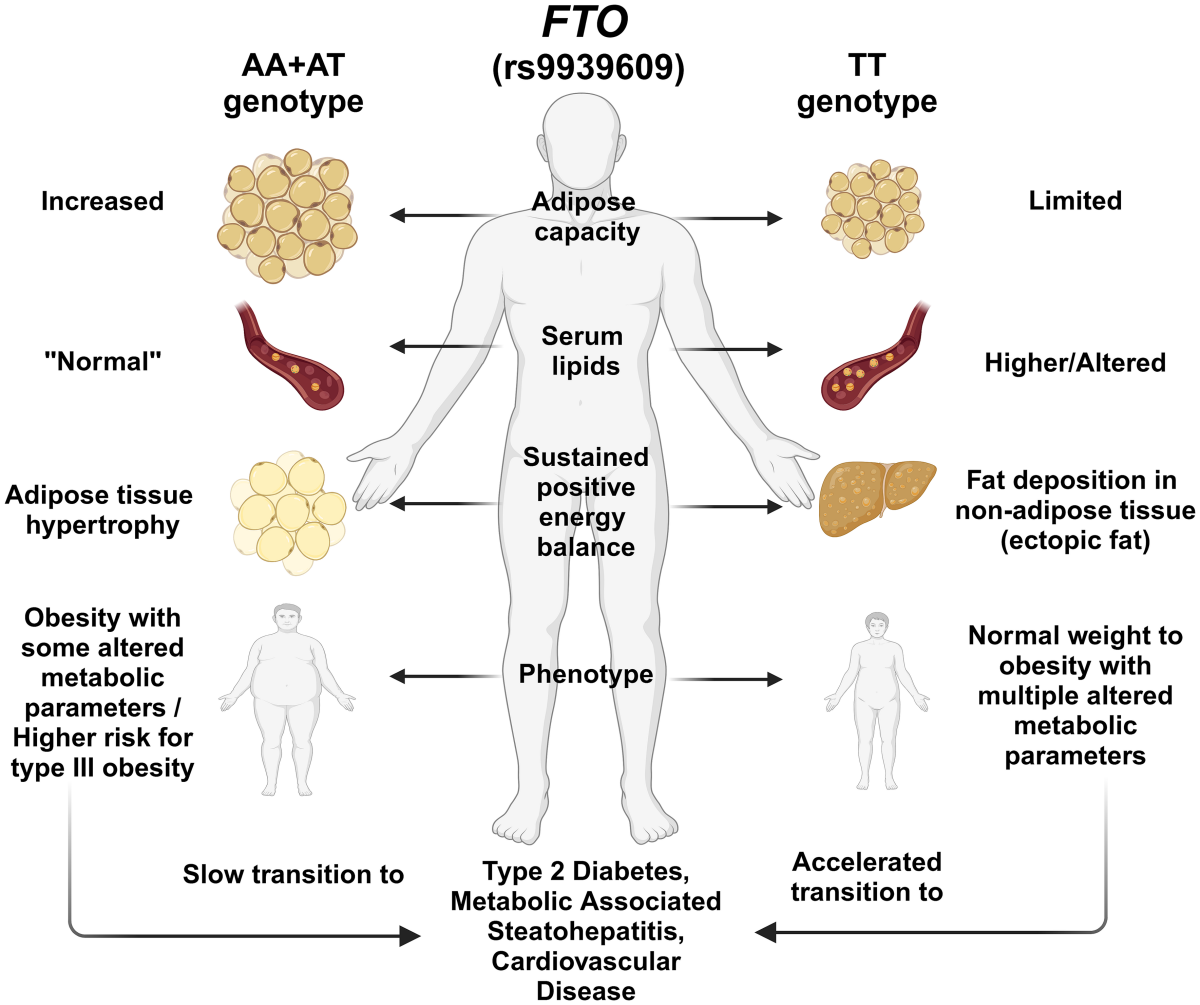
Illustration of the hypothesis of the effect of *FTO* rs9939609 polymorphism genotypes on body fat deposition and metabolic profile. T allele carriers may be at higher risk for metabolic abnormalities associated with T2D and MASLD, possibly due to reduced adipose storage capacity. In contrast, A allele carriers have better adipose storage capacity, which could make them less prone to metabolic disorders. Among the Mexican population, the T allele is predominant among the Amerindians and the admixed population of Guadalajara. In contrast, the A allele prevails more among the Mestizo-Caucasians. This feature could lead to a differential effect of the *FTO* polymorphism among the population.

Therefore, based on the high frequency of the *FTO* (rs9939609) TT genotype among the Wixárika and Nahuas, we suggest that this genotype could contribute to the Amerindian phenotype, characterized by a leaner body, lower adipose capacity, and lower risk of obesity/metabolic disturbances in an ancestral healthy cultural-food environment (Figure 3). Nonetheless, under a modern Westernized lifestyle, with energy-dense diets and low physical activity, adipose capacity is exceeded, contributing to the risk of developing metabolic abnormalities and chronic diseases due to a mismatched gene–environment interaction (Figure 3). Recently, in a cross-sectional study among young university students, an association between the TT genotype and the appetite trait “emotional undereating” was reported, which may reflect an evolutionary homeostatic response to ambient stress (52). In contrast, AA + AT genotypes, which were more prevalent in the Mestizo-Caucasians subpopulations, may be more representative of the European/African population’s phenotype since they are characterized by higher white adipose tissue storage capacity related to different grades of obesity before developing metabolic disturbances (Figure 3). Further studies are warranted to decipher this ethnicity-based association between the *FTO* polymorphisms and environmental factors.

Despite the novel insights, one of the main limitations of this study is its cross-sectional design, which restricts the ability to infer causality between *FTO* genotype and metabolic alteration. Although significant associations were observed, future studies need to consider potential confounding factors that may independently influence metabolic disturbances and interact with genetic susceptibility. These factors include physical activity, sleeping habits, and socioeconomic status, among others. Another discrepancy between our findings and previous studies associating the A allele and obesity is the differences in study design, statistical power, sample composition, and population characteristics that include more individuals with class III obesity (29, 30, 49, 66).

Nonetheless, this study revealed the varying range of the T allele frequency consistent with the ancestral components of the Mexicans and several metabolic abnormalities among the Mestizo-GDL that may be attributed to the Amerindian the T allele component combined with lifestyle factors. These results have significant implications for public health strategies in Mexico, suggesting tailored approaches involving personalized medicine and nutrition that consider genetic differences, lifestyle choices, and cultural preferences (50).

Although one single polymorphism cannot determine an entire phenotype, it can offer guidance for interpreting genetic variants within a specific population under specific environmental conditions from a personalized medicine perspective (33, 67). This is a breakpoint to consider how the mismatch of our ancestral genome to our modern environment could potentially enhance the prevalence of obesity and metabolic abnormalities, which contribute to the leading causes of mortality among the Mexican population. Further investigation into the underlying mechanisms and pathways influenced by the *FTO* (rs9939609) TT genotype can offer opportunities for personalized medicine and nutrition strategies aimed at mitigating and preventing the development of metabolic dysfunction-related diseases in Mexico.

## 5 Conclusion

The *FTO* T (rs9939609) allele was prominent among Amerindians, whereas the A allele prevailed among the Mestizo with higher European ancestry. Mestizo-GDL TT genotype carriers had higher odds of IR, HTG, and HGL, highlighting the genetic predisposition to T2D and MASLD in these populations exposed to obesogenic and hepatopathogenic environments.

## Data Availability

The original contributions presented in the study are included in the article/Supplementary material, further inquiries can be directed to the corresponding author.
